# Level of Physical Activity, Sedentary Behavior, and Sleep in the Child and Adolescent Population in the Autonomous Community of the Basque Country (6-17 Years Old): Protocol for the Mugikertu Study

**DOI:** 10.2196/31325

**Published:** 2022-03-11

**Authors:** Arkaitz Larrinaga-Undabarrena, Neritzel Albisua, Xabier Río, Garazi Angulo-Garay, Xabier González-Santamaria, Iker Etxeberria Atxa, Gorka Martínez de Lahidalga Aguirre, Malen Ruiz de Azua Larrinaga, Aitor Martínez Aguirre-Betolaza, Ilargi Gorostegi-Anduaga, Sara Maldonado-Martín, Juan Aldaz Arregui, Myriam Guerra-Balic, Mikel Bringas, José Ramón Sánchez Isla, Aitor Coca

**Affiliations:** 1 Faculty of Education and Sport University of Deusto Bilbao Spain; 2 Department of Physical Activity and Health Osasuna Mugimendua Kontrola SL Mugikon Bilbao Spain; 3 Mondragón Unibertsitatea Arrasate Spain; 4 Athlon Cooperative Society Arrasate Spain; 5 Faculty of Education and Sport University of the Basque Country (UPV/EHU) Vitoria Spain; 6 Faculty of Law University of the Basque Country (UPV/EHU) Vitoria Spain; 7 Faculty of Psychology, Education and Sport Sciences - Blanquerna, University Ramon Llull Barcelona Spain; 8 Directorate of Physical Activity and Sports Basque Government Vitoria Spain; 9 Health and Consumption Department Bilbao Council/Ayuntamiento de Bilbao Bilbao Spain; 10 Faculty of Health and Sciences University of Deusto Bilbao Spain

**Keywords:** physical activity, sedentary behavior, sleep, Basque Autonomous Community, accelerometry, adolescents, children, healthy behavior, mobility

## Abstract

**Background:**

Physical inactivity and sedentary behavior are increasingly common problems in the general population, which can lead to overweight, obesity, diabetes, cardiovascular disease, and decreased motor and cognitive capacity among children and adolescents. Establishing healthy habits in childhood on the basis of the World Health Organization’s 2020 Physical Activity Guidelines is essential for proper physical, motor, and cognitive development.

**Objective:**

The primary aim of this study is to describe the level of physical activity (PA), sedentary behavior, and sleep of the child and adolescent population from 6 to 17 years of age in the Basque Autonomous Community (BAC). Our secondary aim is to establish a starting point for future research and intervention protocols to improve the existing reality.

**Methods:**

This cross-sectional study aims to recruit 1111 children and adolescents, aged 6 to 17 years from the BAC in a representative random sample. Participants will wear the ActiGraph WGT3X-BT triaxial accelerometer for 7 consecutive days in their nondominant wrist, and fill out a habit diary log of PA, mobility, and sleep routine. PA intensities, sedentary behavior, and sleep parameters (total bedtime, total sleep time, and sleep efficiency) will be calculated from raw accelerometer data using SPSS (IBM Corp). Participants will be randomly selected.

**Results:**

The results of this study intend to demonstrate significant differences in PA levels in different age and gender groups since the volume of school PA in the BAC decreases as the age of the schoolchildren increases. The total study sample includes 1111 participants. In April 2021, up to 50% of the sample size was reached, which is expected to increase to 100% by April 2022. This sample will allow us to analyze, discuss, compare, and assess the reality of the school population, in a sensitive period of adherence to behavior patterns, using data from the geographical and administrative area of the BAC. This study will provide a realistic insight into PA levels among children and adolescents in the BAC. It will also offer scientific contributions on the positive relationship between PA levels and sleep quality in this population.

**Conclusions:**

This study might highlight the need for the promotion of cross-sectional policies so that children and adolescents may increase their levels of PA, thus improving both the school environment and positive healthy behavior.

**Trial Registration:**

ISRCTN Registry ISRCTN65573865; https://www.isrctn.com/ISRCTN65573865

**International Registered Report Identifier (IRRID):**

DERR1-10.2196/31325

## Introduction

### Background

Physical inactivity (PI) and sedentary behavior are highly prevalent around the world and are associated with high morbidity and mortality [1]. In recent years, there has been an increase in the levels of sedentary lifestyle among the child and adolescent population, which is associated with lifestyle and has an impact on increasing obesity, diabetes, cardiovascular disease, cognitive capacities, and mental health [2,3].

In children and adolescents, physical activity (PA) improves physical fitness (both cardiorespiratory and muscular conditions), cardiometabolic health (blood pressure, dyslipidemia, obesity, glucose, and insulin resistance), bone health, cognitive ability (academic performance and executive function), and mental health (reducing symptoms of depression) [2,4,5].

The World Health Organization (WHO) recommends that children and adolescents between 5 and 17 years of age engage in at least an average of 60 minutes of PA per day, involving moderate to vigorous intensity (MVPA), mainly aerobic PA, with a minimum of 3 days a week of vigorous-intensity PA to limit sedentary behavior [2].

In Europe, approximately 80% of children and adolescents do not comply with the daily PA recommendations indicated by the WHO [6-8]. Hence, PI and sedentary lifestyle require intervention from an early age. If not, it may be too late as PA behaviors established in youth are maintained in adulthood [9]. It is estimated that in 2013, PI cost global health systems US $53.8 billion in direct health care, of which US $31.2 billion was allocated to the public sector, US $12.9 billion to the private sector, and US $9.7 billion for households [10]. However, it seems clear that intervention to promote PA must take place at an early age, since establishing habits and behavior changes are much easier and more achievable in childhood and adolescence than in adulthood [11].

In addition, PA is bidirectionally related to a shorter duration of sleep and an improvement in sleep efficiency, since PA contributes to an increase in sleep patterns [12]. Hence, physical exercise is already starting to be prescribed to improve sleep quality [13,14]. However, more national- and international-level studies are required among children and adolescents with objective measures to corroborate these findings [12].

Interventions promoting PA among children and adolescents must be supported by evidence that provides us the necessary information to do so efficiently. This study will allow us to analyze different realities (at different times, school settings, and ages), ultimately facilitating the design of policies and programs to promote PA and health on the basis of stated evidence [15]. Moreover, in the Basque Autonomous Community (BAC), there is a lack of evidence regarding the level of PA among children [16].

To determine the level of PA in the school population aged between 6 and 17 years in the BAC, and considering the benefits of PA for children and adolescents [17,18], this protocol aims to determine whether this group meets the established parameters of daily PA recommended by the WHO [2]. We hypothesize that >50% of the children do not meet the WHO recommendations, and that the level of PA in boys is greater than that in girls.

### Objectives

The main objective of this study is to describe the level of PA, sedentary behavior, and sleep of the child and adolescent population aged 6 to 17 years in the BAC.

The secondary objectives of this study are to (1) contribute to the theoretical, conceptual, and methodological development of research devoted to the study of healthy behavior and well-being of school-age children; (2) monitor and compare healthy behaviors and characteristics of the social contexts in which school-age children develop; (3) develop collaboration with organizations and associations to activate initiatives aimed at promoting health among the school population; (4) promote and support the creation of a network of local professionals to integrate active and healthy behaviors during childhood and adolescence; and (5) strengthen the international research network in the field of promoting PA among school-age children.

## Methods

### Methods Overview

This protocol shows potential in terms of collaborative, interorganizational, and collaborative work among the 3 Basque universities (Basque Country University, Mondragon University, and University of Deusto) along with 2 companies (Athlon Cooperative Society and Osasuna Mugimendua Kontrola Ltd). They are innovative and proactive in the field of PA program intervention for health among different groups and social strata, creating new educational programs at the universities as well as new socio-sanitary interventions prescribing PA for different populations. Thus, they work for transversal and interdepartmental support of the PA and sports, educational innovation, and public health departments of the Basque government.

### Participants and Selection Criteria

According to data extracted from the Basque Statistics Institute (EUSTAT) [19], as of October 16, 2020, the reference population to extract the sample residents in the BAC, aged between 6 and 17 years (born between 2003 and 2014), is 254,093 people, of whom 130,645 are boys and 123,448 are girls.

The corresponding sample size, considering that we are referencing a universal population greater than 100,000 people (referred as infinite for sample size calculation) is 1111 participants, at a confidence level of 2σ of 95.5%, an error limit of 3%, and heterogeneity of 50% [20]. To ensure a proportionate distribution of the sample in all age groups, territories, public and private centers, as well as girls and boys, these 1111 people were selected in accordance with the distribution detailed in Table 1. This sample is defined for all the assessment tests that comprise the project.

There is a proportional and random stratification based on province and county, age, gender, education network (public or private), and medea (socioeconomic level based on the deprivation index per census section, which enables the identification of sections with socioeconomic conditions), along with some inclusion and exclusion criteria that are listed in Textbox 1. Schools were selected randomly on the basis of the above-mentioned criteria.

**Table 1:** Sample size and the current sample (N=1111).

Grade	Araba province				Bizkaia province				Gipuzkoa province				Total			
	Male		Female		Male		Female		Male		Female		Male		Female	
	Sample, n	Current sample, n	Sample, n	Current sample, n	Sample, n	Current sample, n	Sample, n	Current sample, n	Sample, n	Current sample, n	Sample, n	Current sample, n	Sample, n	Current sample, n	Sample, n	Current sample, n
First primary	8	9	7	8	22	6	21	6	14	1	14	1	44	16	43	15
Second primary	7	8	7	8	22	5	21	5	15	1	14	1	44	14	42	14
Third primary	8	8	7	7	23	5	22	5	16	1	15	1	48	14	44	13
Fourth primary	8	8	8	8	24	6	23	6	16	1	16	1	48	15	47	15
Fifth primary	8	6	8	7	24	6	23	6	17	0	16	1	49	12	47	14
Sixth primary	8	6	7	6	24	6	23	8	17	1	16	0	49	13	45	14
First secondary	8	7	7	6	25	4	24	5	17	0	16	1	50	11	47	12
Second secondary	7	6	7	6	25	4	22	5	17	1	16	0	48	11	46	11
Third secondary	7	8	7	7	24	4	22	10	17	0	16	1	48	12	45	18
Fourth secondary	7	8	7	8	24	10	23	8	17	1	16	0	47	19	45	16
First high school	7	8	7	7	24	8	23	14	16	1	16	1	48	17	45	22
Second high school	7	8	7	7	24	3	22	3	17	1	15	1	48	12	44	11
Total	90	90	86	85	285	67	269	81	196	9	186	9	571	166	540	175

**Inclusion and exclusion criteria.**

**Inclusion criteria:**

- Belonging to the student body of a participating school or institute
- Having the authorization to participate through a signed informed consent form by the parents or legal guardians of the child or adolescent

**Exclusion criteria:**

- Nonconsent or refusal by the child or adolescent to complete the physical activity diary or use the accelerometer, even with signed informed consent by their parents or legal guardians
- Physical or intellectual disability that prevents completing the daily physical activity or use of the accelerometer in accordance with the defined protocol. Each case will be assessed with each school’s teaching team and with the parents or legal guardians of the minor

**Figure 1:**
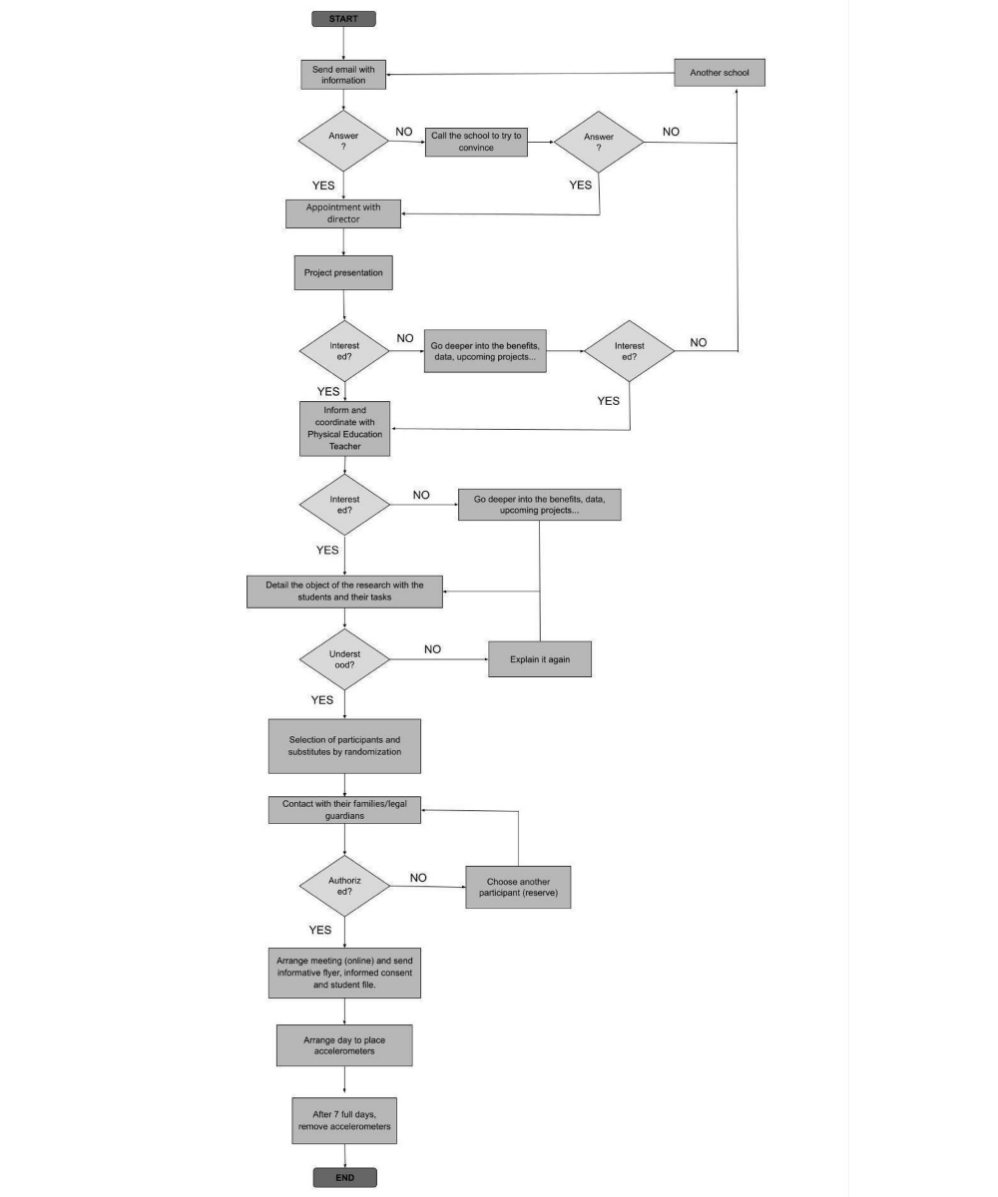
Flow chart of the study.

To reach 1111 individuals, the sample unit selection will be conducted in multiple stages, so that a random sample of all age strata will be obtained, and a subsequent selection (by randomization) will be made to meet the needs of age, gender, and type of established center for the project. The recruitment process can be divided into three phases: contact with the center, contact with the families, and contact with the students. The contact with the centers is initiated via email or a telephone call. If the school shows interest, we arrange a meeting with the school’s principal. The principal selects a person from the school who then becomes the person in charge of the selection process and sending the information to the parents, usually the physical education teacher. Depending on the requirements of the total sample, the age ranges that need to be covered in each school are selected. During this phase, the physical education teachers of each center will be contacted, as well as the counselors for each grade level to request their collaboration and thus carry out an initial preselection of students to participate in the study (Figure 1). The information and the documents to be filled out are sent to the parents. A lottery is held among the participants who meet the requirements (age, gender, and others) and whose parents have given their consent. If the principal, the physical education teacher, the participants, or their parents have queries about the study, they are clarified. A day is set for placing the accelerometers on the selected participants, and after 7 full days they are removed.

### Instrument

ActiGraph WGT3X-BT accelerometers were chosen to measure MVPA and sedentary time behavior. ActiGraph sensors have been widely studied and have shown adequate reproducibility, validity, and feasibility for children and adolescents [21].

Participants will wear the ActiGraph WGT3X-BT accelerometer for 7 full, consecutive days, including weekdays and weekends. It will be worn on the wrist of the nondominant hand. This technique is the most reliable method to record and store the amount of PA quantitatively, allowing us to determine and compare levels of PA demonstrated by each individual in a given period [4,15]. In addition, like other authors, diary recordings will be collected with data such as sleep time, mode of transportation to school or other places, and the kind of PA they engage in [22-24].

To conduct this protocol, 50 ActiGraph WGT3X-BT accelerometers were purchased along with the ActiLife 6 ActiGraph PA software. All the accessories necessary for its use were also purchased, including Velcro wrist straps and cables for data transfer. The accelerometer will be worn on the nondominant wrist for 7 full, consecutive days (worn on day 1 and removed on day 8) including weekdays and weekends. The accelerometer consists of a triaxial activity monitor to capture and record continuous, high-resolution PA and sleep and wake information that provides the following measurements: acceleration, energy expenditure, steps, PA intensity, body position, and lighting ambient.

### Data Collection

Each participant will be identified by a unique and confidential numerical code, which is assigned after informed consent is obtained from the legal guardians. Those responsible for the database must save the files on servers with a password. To determine the codes, the following process is followed:

- Province: 1=Araba, 2=Bizkaia, and 3=Gipuzkoa.
- Town (000)—each town is assigned a 3-digit number (eg, 001, 002, etc)
- Education center is assigned a 3-digit number (eg, 001, 002, etc).
- The academic stage between primary, secondary, or high school or secondary vocational training (1, 2, and 3)
- The grade
- The classroom in letters A, B, C, and D
- The student number (00) considering that each classroom will have a maximum of 30 students.

Consequently, each participant will have a 12-digit code; for example, 2 022 060 11 D 07.

Prior to data collection, the study presentation brochure was emailed to the centers inviting them to participate, along with the informed consent document to authorize their children to participate in the study. Each center shares information about the study with the families through the established communication channel in each case (web platform or email), sending the legal guardians the information received at the center.

If families are interested in participating, they must send their informed consent to the head of the education center, and depending on the number of volunteers, a random draw will be held until the sample requirements established in each area and center are reached.

### Programming and Downloading Accelerometry Data

The computer operating system, compatible with ActiGraph software, has the software license to download the data. Before starting data logging with the accelerometer, each device will be turned on or programmed for data logging.

To program the accelerometer, the participant’s date of birth, gender, height (cm), body mass (kg), and dominant hand must first be recorded. These data will be requested after receiving informed consent. It will be important to inform the participant how to wear the accelerometer before putting it on. The accelerometer can be placed either in contact with the skin or over a sleeve in case of allergies by touch with the parts of the device [25].

The accelerometer is placed on the nondominant wrist without being too tight so that it is not uncomfortable, nor so loose that it moves around the wrist. The part of the button to close the band should be facing toward the fingers of the hand (as if it were a watch). In addition, instructions with photographs will be sent to explain the placement of the accelerometers. The accelerometer should be worn all day and night and should only be removed during activities where the accelerometer could be submerged in water (bathing, showering, water activities, etc). In these situations, it must be removed and put back on the same wrist after the activity is finished.

During the week that the accelerometer is worn, participants must complete a diary to record their hours of daily activity: the time they wake up, nap, sleep, mode, and duration of travel to class, mode and duration of travel to other places, physical education class, scheduled and unscheduled PA, the time when the accelerometer was removed, and circumstances that may have affected their sleep/wake pattern (travel, illness, etc).

With the youngest children, it is important to understand that the diary will often be completed by the parents, guardians or even their teachers. Similarly, from the age of 8 years, children should be encouraged to help their parents or guardians to complete the diary. However, adolescents will be encouraged to complete their diaries themselves, as their parents are often unaware of their sleep routines [25].

Once the full 7 days have elapsed, on the agreed upon date, the accelerometer will be delivered to the person in charge (the teacher or researcher) along with the daily activity log sheet, only including the participant´s code.

Once the accelerometer and the daily activity record sheet have been collected, the data will be downloaded [26]. As this study is conducted with children, the originals will be converted to 60 seconds to calculate sleep [27] and to 5 seconds for daily PA [27,28].

### Study Variables

The following study variables will be recorded: (1) active time and sedentary behavior (in the overall school schedule, physical education classes, school recess time, activities related to school sports and other organized and unorganized exercise, the rest of the time during the day while awake, school days, nonschool days, bouts of light PA, moderate PA, vigorous PA, and MVPA); (2) time asleep (school days, nonschool days, and bouts of sleeping time); (3) type of transportation to school (bus, car, motorcycle, or any other means that do not involve active travel, and time or day of using transportation); (4) type of transportation to places other than school (bus, car, motorcycle, or any other means that do not involve active travel, walking, cycling, or skating); and (5) grade students are attending, BMI, type of centers, location, and socioeconomic status.

### Ethical Considerations

This study was approved by the Euskadi Drug Research Ethics Committee (Basque Government Department of Health) in accordance with law 14/2007 on biomedical research, ethical principles of the Declaration of Helsinki, and other ethical principles and applicable legislation with internal code PI2020011. In turn, current regulations on the protection of personal data will be followed: Regulation (EU) 2016/679 of April 27, 2016 (GDPR), Organic Law 3/2018, of December 5 on Personal Data Protection and guaranteeing digital rights (ES), and Royal Decree (ES) 1720/2007 of December 21.

### Statistical Analysis

The statistical analysis will be performed using SPSS (version 27.0.1.0; IBM Corp). Parametric tests will be performed after all assumptions for each test are met. For comparison between groups (girls vs boys, age groups, school types, and regions) 2-tailed *t* tests, 1-way analysis of variance, or the nonparametric method of Kruskal-Wallis and chi-square tests will be used for the main primary outcomes as dependent variables.

Descriptive statistics will be used for all outcome variables, and the effect size and the level of significance corresponding to the main group (between participants) will be reported. To prevent a type I error, post hoc comparison will be performed when a significant interaction effect is present. Values will be expressed as mean (SD). The significance level will be set at 95% (α=.05).

## Results

The study began in September 2020, and the data collection will take place for the next 2 academic years (2021-2022). To obtain the total study sample (N=1111), 175 participants will be required in the Araba province, 554 in the Bizkaia province, and 382 in the Gipuzkoa province. In addition, these variables are subject to gender, age, center (public or private), location, and socioeconomic status.

There is currently a total of 341 samples among the three historical territories (Table 1). It is estimated that by the end of December 2021, up to 75% of the sample size will have been reached, with 100% potentially reached by April 2022. The impact of the current COVID-19 pandemic has resulted in schedule delays.

## Discussion

### Expected Findings

This study aims to provide current estimates of the levels of PA, sedentary behavior, and sleep among children and adolescents in the BAC (6-17 years old). Sedentary behavior is fundamentally important in the health of children and adolescents [29], and it is highly influenced by the social environment in which they live [30]. Recent statistics show that 28% of children in Spain between the ages of 3 and 8 years are overweight, and 9% are obese [31]. It is clear that increased PA and reducing sedentary behavior play a leading role in preventing overweight and excess adiposity, even when people present an adverse genetic condition that predisposes them to be obese [32]. Some studies seem to have shown an inverse relationship between MVPA with adiposity and cardiometabolic risk, and a positive relationship between MVPA with cardiorespiratory fitness and total body bone mineral density among children and adolescents [33]. Other studies maintain that screen time has a significant and inverse relationship with hours of sleep, unregulated activity (games and other activities), and physical exercise [34].

The results of this study intend to demonstrate significant differences in PA levels in different age groups since the volume of school PA in the BAC decreases as the age of the schoolchildren increases. In terms of gender, it is higher among boys than in girls, reaching the same conclusions as other authors [35,36].

The results will be used to analyze and assess the relevance of creating a battery of assessment of physical condition in the Basque Government (interdepartmental) as an improvement in the diagnosis of the state of physical condition and to be able to intervene from the department of education and the department of health to address, together with the Directorate of Physical Activity and Sport the concrete actions to be developed as process improvement and move in an improvement of physical conditions in future evaluations.

While there are some limitations, such as the current COVID-19 situation affecting the ability to recruit schools, this study has the potential to determine current levels of PA, sedentary behavior, and sleep of children and adolescents in the BAC. Comparing PA levels extracted in situations with and those without COVID-19 restrictions (with or without structured school sports, with or without the geographical limitation of municipal mobility, and with or without structured federated competition among other states that will affect the measurements gathered) can be established.

This study will make it possible to obtain data, analyze them, and discuss various options to promote cross-sectional policies so that children and adolescents increase their levels of PA, improving both the school environment and positive behavior. In turn, this can improve students’ academic results [37]. The results of this study will provide a realistic insight into PA levels among children and adolescents in the BAC. In addition, our results aim to offer a scientific contribution to the positive relationship between PA levels and sleep quality in this population.

### Future Prospects

This study will enable further research on mobility and PA levels among different ages, examine, and analyze intervention protocols to improve health at education centers, and continue studying the relationship between PA levels and sleep in school-age children, in addition to looking for differences between PA levels and race.

### Conclusions

This study might highlight the need for the promotion of cross-sectional policies so that children and adolescents may increase their levels of PA, improving both the school environment and positive healthy behavior.

## Abbreviations

BAC: Basque Autonomous Community
MVPA: moderate to vigorous physical activity
PA: physical activity
PI: physical inactivity
WHO: World Health Organization
